# Ethical implications of epigenetics in the era of personalized medicine

**DOI:** 10.1186/s13148-022-01263-1

**Published:** 2022-03-25

**Authors:** Josep Santaló, María Berdasco

**Affiliations:** 1 Facultat de Biociències, Unitat de Biologia Cel·lular, Universitat Autònoma de Barcelona, Barcelona, Spain; 2 Cancer Epigenetics and Biology Program (PEBC), Bellvitge Biomedical Research Institute (IDIBELL), Barcelona, Catalonia Spain; 3 Epigenetic Therapies Group, Experimental and Clinical Hematology Program (PHEC), Josep Carreras Leukaemia Research Institute, Badalona, Barcelona, Catalonia Spain

**Keywords:** Epigenetics, Ethics, Autonomy, Privacy, Responsibility, Social justice, Personalized medicine, Stigmatization

## Abstract

Given the increasing research activity on epigenetics to monitor human diseases and its connection with lifestyle and environmental expositions, the field of epigenetics has attracted a great deal of interest also at the ethical and societal level. In this review, we will identify and discuss current ethical, legal and social issues of epigenetics research in the context of personalized medicine. The review covers ethical aspects such as how epigenetic information should impact patient autonomy and the ability to generate an intentional and voluntary decision, the measures of data protection related to privacy and confidentiality derived from epigenome studies (e.g., risk of discrimination, patient re-identification and unexpected findings) or the debate in the distribution of responsibilities for health (i.e., personal versus public responsibilities). We pay special attention to the risk of social discrimination and stigmatization as a consequence of inferring information related to lifestyle and environmental exposures potentially contained in epigenetic data. Furthermore, as exposures to the environment and individual habits do not affect all populations equally, the violation of the principle of distributive justice in the access to the benefits of clinical epigenetics is discussed. In this regard, epigenetics represents a great opportunity for the integration of public policy measures aimed to create healthier living environments. Whether these public policies will coexist or, in contrast, compete with strategies reinforcing the personalized medicine interventions needs to be considered. The review ends with a reflection on the main challenges in epigenetic research, some of them in a technical dimension (e.g., assessing causality or establishing reference epigenomes) but also in the ethical and social sphere (e.g., risk to add an epigenetic determinism on top of the current genetic one). In sum, integration into life science investigation of social experiences such as exposure to risk, nutritional habits, prejudice and stigma, is imperative to understand epigenetic variation in disease. This pragmatic approach is required to locate clinical epigenetics out of the experimental laboratories and facilitate its implementation into society.

## Background

Despite the best efforts of healthcare professionals to treat human diseases such as cancer or chronic diseases, the current public health model of post-diagnostic management is unsustainable. Most of clinical units are already unable to cope with the number of newly diagnosed patients and struggle to offer optimal care to manage disease. A paradigm shift is required where a specific individual could be identified and managed not in a community-based environment but under the so-called *personalized medicine* (or *precision medicine*). Indeed, the World Health Organization is committed to foster the implementation of personalized medicine in translational research and health systems for better diagnostics and in the follow-up of citizens and patients [1]. The use of state-of-the art technology, especially the *omics (i.e., genome-wide genetic information or full transcriptome analysis), has facilitated a better understanding of the molecular basis for main human disorders. Not only that, but there is an increased knowledge on how environmental factors influence disease development and progression, being epigenetic factors at the forefront of the molecular links between disease and their influencing factors. At present, the accumulated molecular knowledge is being transformed towards a strategy for a better clinical decision-making and the development of new therapeutic paradigms.

Following from this matter, a tremendous progress related to the scientific knowledge on epigenetic contribution and its development toward translational research leading to implementation in the clinic has being developed in the last years [2]. The reversible nature of epigenetic factors has opened up exciting opportunities to revert aberrant epigenomes, and consequently, the field of epigenetic-based drug discovery has generated several small- molecule inhibitors that are already in clinical practice or under clinical trials (e.g. the DNA methyltransferase inhibitors azacytidine or decitabine, or the histone desacetylase inhibitors vorinostat, romidepsin, belinostat or panobinostat, among others) [3, 4]. Similarly, multiple epigenetic biomarkers such as those predicting response to lifestyle intervention or disease diagnosis are now on the market. The global epigenetics market size is expected to reach 22.05 billion (USD) by 2025, according to a new study by Grand View Research.

Cancer epigenetics is the spearhead for the potential applications of epigenetics in clinical management with epigenetic-based biomarkers successfully proven in cancer diagnosis, prediction of tumor progression and prediction of therapeutic response [2]. Epigenetic biomarkers also possess the potential to be used as screening tools including tests for colorectal cancer (e.g., detection of the CpG methylation levels at septin 9 gene [5] or vimentin [6]) and can act as predictive markers for estimating the response to chemotherapy (e.g., MGMT promoter hypermethylation in glioblastomas [7]). Epigenetic abnormalities have been also detected in common neurological diseases, such as amyotrophic lateral sclerosis, Parkinson’s disease or Alzheimer’s disease as well as various psychiatric diseases including schizophrenia, major depressive disorder or posttraumatic stress disorder [8]. Although the number of preclinical studies on the potential biomarker use of epigenetic alterations in neurological and neurodegenerative disorders is increasing (e.g., α-synuclein methylation in Parkinson’s disease [9] or promoter methylation in genes associated with deregulation of the amyloid-β (Aβ) peptide in Alzheimer’s disease [10, 11]), they are still not implemented into clinical practice. Technical limitations and lack of appropriate in vivo models are limiting factors to the implementation of epigenetic-based biomarkers in neurological disorders.

**Fig. 1:**
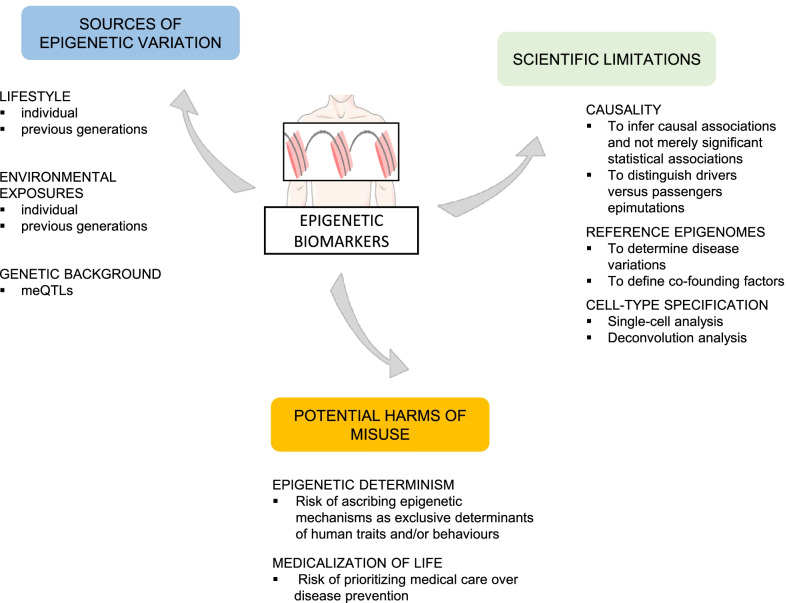
Epigenetic-based biomarkers to monitor human diseases. The effects of lifestyle, environmental exposures (at individual or transgenerational level) or the genetic background, among others, are well-known influencing factors of the epigenome. In spite of the growing number of proposed biomarkers associated with human diseases, some technical limitations need to be solved including the assessment of causality, the establishment of reference epigenomes or the cell-type specificity. On the ethical, political and social dimension a deep discussion on the role of epigenetics as determinants of health and the impact of public health policies and personalized medicine is required

Undoubtedly, the flexibility of the epigenome has generated another enticing strand of studies to understand how epigenetic changes (associated with disease) can be linked to lifestyles and environmental exposures, including diet, physical activity, tobacco smoking, alcohol consumption, exposure to chemical or physical agents or psychological stress, among others (Fig. 1) [12]. The question is: Could epigenetic information be used to monitor lifestyle interventions on patients designed to reduce disease risk or progression? Metabolic diseases including type-2 diabetes [13] and obesity [14] are at the forefront of such approaches. Patients suffering from type-2 diabetes respond differentially to exercise programs, and their responses are associated with promoter methylation of the PPARGC1A gene which encodes for a protein involved in the control of glucose and fatty acid metabolism [13].

Given the increasing research activity on epigenetic-based biomarkers together with the connection with environmental conditions and lifestyle and its reversible nature, the field of epigenetics has attracted a great deal of interest in both the social sciences and humanities. The discussion of whether epigenetic data pose important new challenges that can lead to an ethical-legal framework very different from that generated by the use of other molecular biomarkers (e.g., genetic data) or if it just introduces an increased degree of complexity in “old” ethical, social and legal issues (ELSI) is a controversial but timely debate [15, 16]. However, although solid scientific evidence has yet to be generated to understand certain epigenetic processes and their association with the disease (especially those related to the influence of environmental factors or transgenerational inheritance, among others), now is the time to anticipate the discussion and identify the risks to propose preventive measures [17, 18].

Repeated questions have arisen in forums for ethical discussion in the last years. Some of the concerns are not exclusive of epigenetic research and are contained in ethical guidelines for biomedical research involving human subjects such as the Belmont Report [19] and the Declaration of Helsinki [20]. However, the complexity of some epigenetic concerns is increased. To mention some examples: given the possibility of evaluating the consequences of lifestyle habits on health through epigenetic biomarkers, where is the limit of individual responsibility for their own or even the next generation’s health? In a hypothetical case where an available epigenetic test to detect that an increased risk of having lung cancer is a consequence of the smoking habits of the patients, should the health system be designed accordingly to the patient’s responsibility on this action? On the other hand, nowadays it is widely accepted that specific environmental toxins—pollution, plastic components, use of pesticides or hormones in food—are known (or under suspicion) to influence disease on-set, at least in part, by epigenetic mechanisms. According to this, the question arises: Who is liable of such expositions? Undoubtedly, policy decision-makers and public policies must acquire an increased responsibility. Assuming that social and/or political structures of the population could influence the risk of epigenetic based diseases—with low socio-economic classes at the most “epigenetically disfavored” situation—these vulnerable populations would be at a higher risk of social discrimination and inequality in the universal access to epigenetic-based medical care.

In this review, we will explore these and others concerns—mainly associated with autonomy, privacy, equal opportunities and responsibilities—in epigenetic research, highlighting the main technological limitations and challenges to be answered before advancing from theoretical to practical dimension. We end with brief comments on how misinterpretations of the epigenetic influence on disease could lead to a way of non-genetic determinism and a reflection on whether epigenetic research has the potential to jeopardise personalized medicine interventions at the expense of reinforcing public policy measures. We have employed a systematic search strategy that utilized the PRISMA Statement to conduct the review [21]. We have conducted searchers on the PubMed and Google Scholar databases for peer-reviewed journal articles published in English. The keywords used as search terms were: epigenetics, ethics, autonomy, privacy, responsibility, social justice, personalized medicine, transgenerational inheritance or synonyms.

## Communicating epigenetic-based risk assessments: which, when and how

As we have a very complex and dynamic epigenome, which depends on the tissue, age, exposure to environmental stimuli, lifestyle or pathological situations, among others, we do not have a unique epigenome during our lifetime [2, 22]. Moreover, it is also difficult to generate a so-called normal epigenome which is comparable to any specific situation (Fig. 1). Consequently, the elaboration of epigenetic maps involves the quantification of multiple situations and the generation of a large volume of personal data that is being accumulated in secure databases [23, 24]. International initiatives to unravel the epigenomes at global level have been launched in the last decades, placing particular attention on the International Human Epigenome Consortium (IHEC), a global consortium with the primary goal of setting up high-resolution reference human epigenome maps for normal and disease cell types [25]. In light of the growing amount of epigenomic research data and health records that are being collected, the IHEC consortium has incorporated the Bioethics Working Group to identify and discuss current and emerging ethical concerns of epigenetics research, and to elaborate guidelines for a better ethical assessment.

**Fig. 2:**
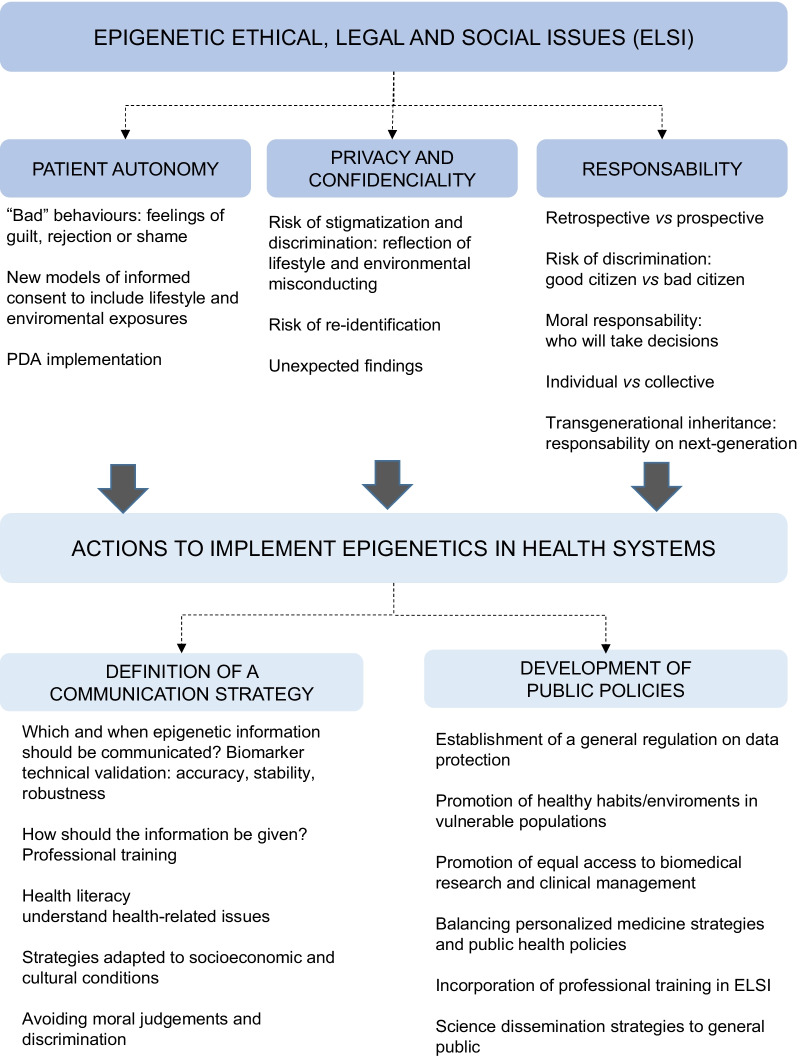
Ethical, legal and social issues (ELSI) and challenges associated with the use of epigenetic-based biomarkers in the management of human diseases. Specific ELSI considerations on patient autonomy, personal data privacy and confidentiality and personal responsibility are derived from the intrinsic epigenetic characteristics (*upper panel*). To tackle these ELSI concerns and to favor the implementation of epigenetic-based approaches in medical care some challenges have been identified. On one side, and given the complexity of epigenetic mechanisms, future communication strategies aimed to ensure the understanding of the epigenetic information during medical decision making are strongly recommended (*down, left*). On the other hand, public policies such as the definition of general laws on data protection, the promotion of equal access to healthy environments and biomedical services to all citizens or the development of educational scientific programs for public but also healthcare professionals are strongly encouraged (down, right). The engagement of the scientific community and health-care professional with policy-decision makers and general public would definitively lead to new biomedical practices and transformative change in health promotion and medical care

Translating complex epigenetic research information for a non-specialized public represents a challenge for communication strategies (Fig. 2). Which epigenetic information should be communicated to the patients? When can a biomarker be considered in the clinical setting? To answer these questions and provide an ethical and legal advice on this matter, the IHEC Bioethics Working Group propose to check the following points previous to communicate epigenetic information [26]: (i) data accuracy. Quality control processes are required (technical validation), and the replicability of the findings should be demonstrated in a clinically accredited diagnostic laboratory before any research results are returned. The origin/source of the epigenetic data, such as the cellular and tissue composition, the age and gender of the individual, needs to be considered; (ii) stability of the epigenetic-based biomarker. Since epigenetic marks are dynamic, does the biomarker remain stable overtime? Epigenetic analysis at different time points is highly recommended; (iii) causality of the epigenetic mark. Is it merely a significant statistical association? Is it a statistically inferred variant? Or are they causal variants where disease causality has been proven? Causal variants are the optimal candidates for clinical validation as a first step towards biomarker actionability; (iv) clinical value of the biomarker. The magnitude of the disease risk and severity as well as the potential to revert epigenetic risk variants through specific treatments should be examined.

Whereas the “*which*” and “*when*” responds to the technical validation of the epigenetic discovery, the “*how*” focuses on the individual. Communicating disease risk/outcome is a difficult and complex process, but undoubtedly, finding the best way to communicate the information to the patient encourages a stronger relationship between health professionals and their patients, a greater confidence in health systems, and may globally improve the quality of health care. Although there is no single formula, there are multiple recommendations aimed to obtain optimal communication practices including facilitating personalized information (e.g., adapted to age and/or sociocultural background) by using presentations in multiple formats, providing honest and objective information, allowing for sufficient time for listening and interpretation, attention to the expression of emotions, and respecting the sociocultural moral values and decisions of the patients, among other recommendations [27]. Public measures aimed to facilitate the professional training in epigenetic and ethical associated matters will improve the communication process (Fig. 2).

The action of communicating an epigenetic-predictive result itself could imply a risk of “burdening” some members of society. This could occur when the possibility to revert the risk is presumed to be dependent on the individual’s behavior, but the individual has reduced effective possibilities to alter his/own behavior or environmental exposures. What may also occur, is that the proposed changes to the individual’s behavior are not followed due to what may be described as a moral weakness of the individual, by the proponent practitioner. One example of this is poor accessibility to sources of healthy food or limited possibilities to move from “unfavorable” environmental exposures to improve the health of vulnerable sociocultural populations. For example, if the epigenetic test reveals a high risk of suffering type-2 diabetes derived from obesity but there are limited possibilities to improve food habits, should the test be communicated? Alternatively, if the patient has a long history of breaching dietary suggestions, is it ethical to communicate to him/her the epigenetic implications of such behavior? A similar situation appears in those cases of patients with high risk or poor prognosis where there is lack of truly effective preventive or curative strategies. Should it be reported in these cases? In summary, knowledge of the degree of risk or disease outcome can lead to the violation of individual rights and promote discriminatory reactions. On the other hand, additional concerns such as patient autonomy or the ethical responsibilities of the healthcare professionals facing non-actionable information should be also considered. The “non-conditioning information” paradigm followed in conventional “genetic counselling” should be seemingly respected to preserve the autonomy of the subject (see “Patient autonomy” section). Like communication of genetic counselling, the ethical obligations aimed to promote the duty of disclosure actionable epigenetic risk may also be applicable in this case. In summary, there are some risks in the communication of epigenetic results that could be balanced by giving appropriate information from epigenetic experts. The creation of specialized services guided to provide “epigenetic counselling” and/or the promotion of genera public dissemination of science should be considered as a tool to facilitate the transmission of epigenetic knowledge to non-epigenetic experts and general public.

## Ethical implications of epigenetic-predictive biomarkers

In sharp contrast with the extensive literature built-up on the ethical implications of genetics, the references to the ethical implications of epigenetics research are less documented although an increasing attention has been paid in the last years. The same cannot be said for the number of ethical concerns associated with epigenetics given that the consequences on epigenetic research could have an impact both at the individual and social level. The reversible nature of epigenetic factors—and the potential to revert or prevent aberrant epigenomes—together with its link to environmental exposures or personal behavior creates a new scenario for debate on environmental justice, social and personal responsibilities on health promotion or intergenerational equity, among others [16, 28].

In this section, we will restrict to the main ethical issues associated with the development and implementation of epigenetic-based biomarkers for assessment of disease risk and/or disease outcome, including (i) patient autonomy; (ii) privacy and confidentiality; (iii) personal responsibility for health; and (iv) justice and equality of opportunities for health promotion (Fig. 2).

### Patient autonomy

Epigenetics provides additional information about lifestyle (e.g. diet, obesity, smoking, hormone supplementation) [14, 29, 30] and the environment (e.g. exposure to endocrine disruptors) [31] to which the patient has been or is exposed. To the extent that the lifestyle could be inferred from the epigenetic state, a change in the principle of autonomy can occur. The knowledge that a “bad” lifestyle inevitably leads us to suffer a pathology when it is in our hands to make it change can generate feelings of guilt, rejection or shame. These feelings can be increased in those cases where there is responsibility for the offspring accordingly with the transgenerational epigenetics concept-defined as the molecular mechanism by which environmental exposures and lifestyle decisions can affect the offspring directly through the gametes or in utero exposures during pregnancy [32].

In a more practical aspect, the inclusion of lifestyle aspects inferred through epigenetic biomarkers should lead to the reconsideration of new models of informed consent. Informed consents for disease risk predictions should be adapted to accommodate for the patient’s age, level of education and engagement. Informed consent should clearly contain the risks and the benefits, the harms associated with the screening, and the existence of alternative prediction methods [33]. The inclusion of lifestyle data, its connections with epigenetics and the consequences of this inference can generate excess information that hinders understanding and, therefore, the patient's ability to identify the relevant information associated with their clinical decisions and an autonomous choice. What does an autonomous choice mean? It means an intentional and voluntary choice taken after an appropriate understanding of the information, and importantly, a decision in line with patient’s personal values [33]. However, the reality at the clinical environment is that there is a saturation of ethical and legal documents (mainly the latter) to which the patient is subjected to [34]. The creation of new informed consent documents adapted to epigenetic research should avoid increasing their complexity and ensure that the basic principles of informed consent are maintained. In this regard, tiered–layered–staged informed consent has been proposed as an appropriate model for epigenetic-based biomarkers [35]. The tiered–layered–staged informed consent, which was originally proposed for commercial personal genome testing [36, 37], is based on three components: (1) *tiered*, meaning that individuals have the option to differentially consent to different parts of the treatment or testing in function of their ethical, personal or social preferences. For example, individuals could give their consent only to perform a predictive test when a clinically actionable target exists [35]; (2) *layered*, as the informed consent model incorporates layers of complexity ranging from minimal information that could be understood by all individuals towards additional layers with more detailed information; and (3) *staged*, which means that the information is given in several stages during the clinical process. In practice, the information is accommodated during all steps of the screening test, and the informed consent is renewed accordingly [36]. Although the tiered–layered–staged model improves how information is distributed to the patient, and could strongly contribute to organize and resolve the complexity and dynamic nature of the epigenetic information, for some authors it lacks the support for the processing of the information on function of the patient’s value [35]. The implementation of Patient Decision Aids (PDA), defined as evidence‐based tools designed to help patients make specific and deliberated choices corresponding to their health decisions that supplements clinicians’ counselling [38], is strongly recommended as a mechanism to include the patient’s own values [35]. Since epigenetics adds uncertainty to the outcome of the disease screening (due to the potential ability of reversion) as well as generates a personal decision about whether a change in lifestyle is possible, the autonomous decision of an individual for being included in disease prediction test acquires an added dimension. Future strategies aimed to promote the informed consent process and the autonomous choice in line with own values should be developed by the health-care system and policy-makers.

### Health information privacy and confidentiality

Privacy and confidentiality have been broadly discussed in the literature on the basis of genetic research [39, 40]. Whether epigenetic research creates a new perspective or it just maintains the same concerns with a (probable) increased complexity is under strong debate [15, 41]. As far as the epigenetic consequences on the privacy and confidentiality of the individual’s health information is concerned, we have, above all, to introduce the definitions. *Privacy* refers to the individuals’ right to control the acquisition, uses or disclosures of their identifiable data relative to health; whilst *confidentiality* defines the obligations of those who receive health information to respect the privacy interests of the data donors [18].

Epigenetic data present the same privacy and confidentiality considerations regarding data security as genetic data [42]. These previous legal and ethical issues on data protection in genetic research offer a guideline to handle those concerns; however, epigenetic information adds an extra layer of sensitivity by the potential ability for containing information about the individual’s previous behavior and environmental exposures that intensify the need for data protection [43]. This information, if not confidentially treated, could promote stigmatization and discrimination of specific collectives [44, 45]. Although the empiric evidence is still a promise, we could anticipate a scenario where participants could enter in an epigenetic- predictive screening to determine whether they have developed epigenetic alterations associated with environmental exposures or lifestyle (e.g., smoking, alcohol or drug abuse) [30]. Participants should have the option to restrict the access of information to third parties, including employers, insurers, family, friends or healthcare providers. An evaluation of the risk is needed before implementation of epigenetic-based screenings in the population, especially in vulnerable groups (e.g. lower socioeconomic groups or ethnicities, among others) to avoid this non-genetic discrimination.

Re-identification of the sample donor is a crucial ethical issue associated with privacy in genetic research [23, 46, 47]. Whether epigenetic information stored in public databases also exposures to genetic information is under strong debate. Specifically, whole-genome bisulfite sequencing (WGBS) to determine genome-wide CpG methylation provides DNA sequence at base-pair resolution. While absolute data confidentiality and privacy cannot be guaranteed regarding high-throughput epigenomic data affecting DNA, some measures have been proposed to mitigate the risk of re-identification [23]. First, removal of indirect genotype information (e.g., single-nucleotide polymorphisms, SNPs) by pre-filtering prior to open-access release using existing algorithms and genotyping resources or masking sites (CpGs or probes) that have common SNPs is recommended. However, following the guidelines of The Cancer Genome Atlas (TCGA) project, somatic genetic mutations could be reported (but not germline mutations) [23]. By contrast, the intrinsic variability of epigenetic markers depending on tissues or age, for instance, may act as a safeguard towards re-identification efforts.

The second aspect refers to how concerned are donors and the general public. It is necessary to improve the communication with the sample donor to facilitate understanding of the risks and benefits of the analysis. Elaboration of standard consent information documents and data-access agreements are highly recommended.

Whether sensitive information concerning lifestyle could be inferred from epigenetic information still needs further studies. In spite that a study reveals that smoking and alcohol consumption could be revealed through DNA methylation data from blood [30, 47], these phenotypic-epigenetic associations have not yet been replicated for additional behavioral habits, and most importantly, whether these hallmarks are persistently maintained in different tissue samples still needs validation. Most probably, debate in this area is mostly anticipatory and speculative [15, 48]. The truth is that we are not facing a novel problem but, rather, a challenge that has long been debated for similar studies involving gene expression datasets [49] or epidemiological data [50].

In addition, a recurrent privacy-related debate exists on the fact that genetic risk predictors could also result in unexpected findings [51]. Epigenetics poses additional challenges derived from its potential to infer lifestyle. As previously mentioned [30], smoking could be associated with specific promoter methylation. What is more, smoking-associated DNA methylation changes (e.g., AHRR or F2RL3 genes) have been described to predict risk of lung cancer [52, 53]. In addition, AHRR methylation has been also observed in carotid intima-media thickness and consequently in cardiovascular risk [54]. In the hypothetical case where a risk prediction test for lung cancer based on AHRR methylation exists, should the incidental finding of an increased risk cardiovascular disease be communicated? In other words, does unsolicited findings need to be communicated? There is not any consensus on this aspect; however, recommendations have been proposed where the bottom line is to evaluate benefits and harms. Additionally, the right of “not to know” of the subject, especially in those cases when no efficient treatment is at hand (as it is currently the case in many epigenetics biomarkers), must be respected.

Finally, it should be highlighted that data protection and privacy are also a responsibility of the policy decision-makers. It is necessary to update the current legislations to adjust the problem frames derived from epigenetic research (i.e., privacy and anti-discrimination laws) and to establish general regulation on data protection; equally important is to configure secure open-access metadata services adapted to privacy standards but allowing an access for the scientific community interested in epigenetic research (Fig. 2).

### Personal responsibility

Two conceptualizations of personal responsibility can be drawn: a backwards-looking notion (or retrospective) and a forward-looking notion (or prospective) [55, 56]. The retrospective vision arises as an interpretation of a detrimental effect as a causal consequence of a bad lifestyle or environmental exposures. In line with this vision, an individual has the possibility to select those behaviors that promote health and to exclude the harmful ones. If so, this individual will be automatically identified as a “good” citizen. On the other hand, a person showing unhealthy habits (e.g., alcohol abuse, smoking, drug consumption) will be stigmatized as a “bad” citizen, increasing the risk of feelings of guilt, blame and also discrimination. As later discussed in this review, caution should be exerted at this point because whether epigenetic modifications are the consequence of voluntary decisions of the individual and the causality of such epigenetic changes on the pathogenesis is still unclear [57]. The real ability of individuals or even their chances to avoid harmful exposures during their lifetime should be placed in context.

The prospective vision of responsibility focuses on the question of who is supposed to take future actions to mitigate the disadvantageous epigenetic effects. Several concerns should be considered in this context of prospective responsibility. Does the certainty of a bad habit really impact our behavior? Supposing that the following situation occurs: there is a validated epigenetic biomarker in clinical use that is responsible for an increased risk of colorectal cancer, and that this biomarker could be reverted to the healthy value with a diet intervention and, consequently, decrease the cancer risk. Undoubtedly, this epigenetic biomarker will create an exceptional opportunity for preventive medicine. However, whether an individual would adopt a change in the lifestyle after obtaining a risk- predictive testing for disease is not well determined [55]. Most of the current examples come from screening tests based on the presence or absence of specific genetic mutations in relevant genes. A systematic review of metadata obtained from multiple controlled trials involving adults that have received personalized high-risk estimations based on their genetic background where risk for disease could be reduced by adopting behavioral changes reveals that communicating the high risk estimated does not promote a healthy behavior to reduce the risk [58]. Although there is still no evidence in epigenetic studies, it should not be expected to be distinct from other molecular tests (e.g., genetic or proteomic-based tests).

One determinant factor in the adoption of a change in the lifestyle after an epigenetic risk assessment is to have competent skills to understand and applied the epigenetic information for a better health. The ability of an individual to access and understand health information in order to take decisions concerning health care, disease prevention and health promotion (named as *health literacy* [59]) strongly varies among populations. A recent study examined the link between health literacy skills and diabetes risk among non-diabetic adults in the German population concluding that low health literacy was associated with behaviors that increase the risk of type-2 diabetes such as smoking, inactive lifestyle and poor dietary habits [60]. Similar correlations have been found for low health literacy and another diseases, including mental illness [61], cancer screening [62] or the COVID-19 pandemic [63], among others. Education level, socioeconomic status and physical limitations to perform routine activities (e.g. limiting chronic diseases) strongly contribute to inadequate or problematic health literacy [64, 65].

The intrinsic complexity of epigenetics could be a barrier for the understanding of the information about how it contributes to the risk for the disease or whether it could be modified by changing our environmental exposures or lifestyles. Accordingly, those groups with low or problematic health literacy would manifest problems to adapt their lifestyle or exposures based on their epigenetic profiles. Even if we assume that an epigenetic biomarker could predict the risk and, most importantly, could be used for monitoring the result of risk- reducing intervention, the potential benefits will not be accessible for the whole population because of inequality in health literacy.

Once again, it must be mentioned that the presumed abilities to change our epigenome by modifying lifestyle and exposure to toxic environments leads to the discrimination or stigmatization in more vulnerable populations. It is questionable how the individuals could really modify their lifestyle towards a healthier situation. The development of public policies aimed to promote healthy habits and environments in the citizens with a special attention to vulnerable populations will facilitate this change. Policies focused on behavior change (individual lifestyle) and policies that either wish to provide people with the resources to make better decisions (e.g. empowering them with more knowledge, more economic resources, etc.), or modify the relevant social configurations that orient these choices (e.g. nudging, taxation, ban of certain products, etc.) have the potential to introduce healthier lifestyles (Fig. 2). Another variable that should be taken into account is the time needed to effectively revert the epigenetic profile towards healthier situation. Does it take few months or an entire lifetime?

Another relevant aspect of this prospective vision of responsibility is related to the transgenerational epigenetic inheritance. In this case, the personal behavior and habits have an influence on the individual offspring, transmitted through the epigenetic profiles of the gametes, generations ahead [66]. Who is responsible for this inheritance and for its quality? Would it be possible that next generations might hold us accountable for our “misconducting” in topics such as pollutants exposure or lifestyle both at individual and collective (social) levels? The ethics related to what kind of world are we going to leave to our descendants may also include the epigenetics of the next generations.

## Does epigenetics provide a bridge between biomedical ethics and environmental ethics?

The inclusion of environmental factors and lifestyle and their influence on health represent a paradigm shift in the search for predictive models of disease. Is epigenetics providing a scientific basis for the transition from individualistic predictive models to collective prediction models? Is the molecular alteration at the individual level the objective of the study or, on the contrary, the environmental factor that brings together a group of individuals the main target? These questions generated by the field of epigenetics reopen an old dichotomy between ethical schools of thoughts in the field of health: Biomedical Ethics and Environmental Ethics [67]. Biomedical Ethics, which mainly represents the North American approach developed around the bioethical principles of Beauchamp and Childress [68], focuses mainly on important ethical dilemmas that occur in health care, in the field of biomedical research and in the use of new biotechnologies in medicine. Environmental Ethics focuses on issues pertaining to the relationship between human activities (including health) and the environment or the social, economic and cultural contexts [69]. Epigenetics, as a molecular explanation between the expression changes of our genes and the external and internal environmental conditions to which we are exposed, represents a great opportunity for the integration of the two conceptualizations of Bioethics.

Exposures to the environment and individual habits do not affect all populations equally, creating a violation of the principle of distributive justice in bioethics. Frequently, the most vulnerable populations are precisely those that live in the most unfavorable environmental conditions and that have fewer economic and cultural resources to mitigate the adverse effects of the adverse agent. To mention an example, the pollution generated by industry is often transported far from the geographic location where it occurs [69]. Therefore, the population that suffers the adverse effects derived from pollution does not coincide with the population that generates the pollution. If that local population does not have infrastructure to overcome it (for example, detoxification of contaminated water), there is a clear injury to the most vulnerable populations who suffer an unfair distribution of risks to potentially alter their epigenome and worsen their health. Given the pressing importance of promoting health care, we envisioned that epigenetic mechanisms should be incorporated into the broader discussions of the social determinants of health inequalities.

## Current ethical and social challenges raised by epigenetics

### Limitations in epigenetic research

Based on all the issues discussed above, it is clear that epigenetic research in the context of disease prediction opens new (or potentiate old) ethical, legal and social concerns. This represents a scenario as a starting point for discussion; however, some of the issues for debate are exploratory because specific barriers in epigenetic research still need to be overcome (Fig. 1) before advancing from theoretical to practical dimension in ethical, legal and social discussion [70, 71].

Causality is the Achille’s Heel in epigenetic research. Bibliography is full of preclinical correlated associations between an epigenetic alteration and disease states; however, whether this association is critical to determine the causality of the disease is poorly understood in most cases. Does promoter methylation of a tumor suppressor gene influence cell proliferation? Or by contrast is the gain of tumorigenic properties a trigger of the promoter methylation? Definitely, one great challenges in clinical epigenetics is to distinguish causal changes, so-called *drivers*, from changes that appeared by chance but did not contribute to the transformed phenotype themselves, so-called *passengers*. This causality acquires an extra level of complexity when associations involved an environmental factor or lifestyle habits. Smoking has been widely associated with epigenetic alterations and increased risk of respiratory diseases (e.g., chronic obstructive pulmonary disease or asthma) or neurodevelopmental disorders (e.g., child attention-deficit/hyperactivity disorder) at a single generation but also in transgenerational inheritance [72, 73]. However, studies involving different human cohorts did not allow for conclusions that a causal association between smoking, methylation and disease output exists [72]. Does additional influencing factor exist? Are methylation levels influenced also by diet, local pollutants or age of the study participants? Are DNA methylation levels affected in a cell-type manner?

To solve part of these questions and address epigenetic causality in basic research, two main strategies are being developed. At one end, it is necessary to consider data integration tools [74, 75]. Undoubtedly, epigenetic research centered on a unique epigenetic modification will not provide a comprehensive functional view of most biological processes and multiple epigenetic marks should be incorporated in the biomedical research to elaborate a closer in vivo scenario. Not only that, -omics integration (i.e., transcriptomics, genomics, epigenomics and proteomics) could contribute to outline the functional role of the epigenetic state. Molecular data integration will depend on the design of appropriate approaches for the standardization, annotation and harmonization of epigenetic data, as well as optimization of computational and machine learning methods [76]. On the other side, unravelling the functional implications of epigenetics in a disease could take advantage of the epigenetic editing tools (e.g., based on CRISPR-dCAs9 technology) [77]. By these tools, it is possible to modify the epigenetic marks at specific loci for the creation of chromatin contexts [78]. By doing so, epigenetic editing allows to establish the functional effect of the epigenetic perturbation using cellular and animal models—beyond inferred clues from computational approaches.

It is equally important to mention the difficulty to establish what a “*healthy*” epigenome means. There are epigenetic variations that confers an advantage for a biological adaptation in response to environmental (internal or external) stimuli. Such natural epigenetic adaptations should not be confounded with epigenetic changes showing a causal role in diseases. Furthermore, the establishment of “*reference*” epigenomes implies to gain knowledge on cell-type heterogeneity [79] and on the natural variations associated with developmental stages, that is to say, to understand the normal dynamism of epigenomes during human cellular differentiation and development [80]. These concerns, and others such as developing human models, should be solved before ascribing moral epigenetic responsibilities as a result of a voluntary lifestyle or environmental exposure.

### Epigenetics: tipping the balance in favor of personalized medicine or public health policies?

As Dupras and Ravitsky note [81], the development of the clinical epigenetics concept, that is to say, the translation of the epigenetic knowledge to the clinical management [2], has the potential to jeopardise public policy measures at the expense of reinforcing the personalized medicine interventions. The difference is the point at which the focus of disease prevention is. If external factors such as socioeconomic, cultural or living environments are considered as determinant for health, epigenetics could provide a molecular mechanism to reinforce the necessity of making “better” external conditions aimed to create “healthier” epigenetic conditions and to reduce epigenetic health inequalities. This reasoning is in line with the implementation of collective preventive strategies at the policy level [81]. On the other side of the coin, the emphasis can be on the internal molecular etiology of the disease and the search either in the prediction of disease risk or progression (biomarker use) or the development of epidrugs to revert aberrant epigenomes. This second vision is aligned with the personalized medicine approaches. In principle, both visions have the potential to positively impact in the healthcare promotion of citizens; however, how to prioritize one strategy represents a conflict because public health resources are limited.

Nowadays, discussion on the balance between benefits and harms of personalized medicine has generated a dichotomy. So far, personalized medicine is based on the stratification of patients based on their molecular profile (mainly but not limited to genetics) combined with artificial intelligence, which leads to a more person-centered heath care in accordance with the “*right treatment for the right person at the right time*” reasoning. A few examples of the implementation of epigenetic knowledge in personalized medicine exist [2]. However, it is true that this number is still too low for considering a relevant impact on public health. Are the benefits of epigenetic personalized medicine overpromised? Some detractors argue that personalized medicine’s effect on public healthcare costs are unclear [82]. First, personalized medicine requires a high investment in -omics technologies that are high-cost consuming. And second, it is unclear whether public health systems would be able to sustain specialized drug treatments for small groups of people that entails a high production expense. The possibility that personalized medicine exacerbate inequality in access to health care is also broadly discussed [83]. The equal access to the benefits of epigenetic-based biomedical research and clinical management needs to be guaranteed by adopting appropriate public policies (Fig. 2).

This discussion turns to whether addressing the underlying causes of the disease, that is to say, disease prevention would be preferable than to create new specialized therapeutic strategies. Where public resources should be mobilized? Epigenetics could establish a bridge between personalized medicine—the molecular level of the disease—and the epidemiological context of the disease or the living conditions. Can epigenetics fill the gap between the personalized medicine and the personalization of health care? [84].

However, due to the lack of robust scientific evidence on the effects of the environment and lifestyle in the epigenome and their result in impaired health, most of the discussion on public health strategies to reduce the incidence of human diseases are not implemented in a practical context. Current experimental models to unravel epigenetic causality of complex diseases linked to environmental and lifestyle expositions have been developed, including the Environmental Enrichment (EE) protocol. EE consist on the optimization of the housing conditions for murine models by providing physical, cognitive, sensorial and social stimulation [85], and it has been used to ameliorate the adverse epigenetic effects associated with various neurological and psychiatric disorders [86]. Whether this EE model, which emphasizes the material, organic and molecular traces of experiences, elevate models of political and collective intervention is under strong debate [87]. Moreover, the personalized medicine strategy is nurtured by the increased acceptance of two social trends: *molecularization* and *biomedicalization* [81]. The first refers to the inclusion in the molecular arguments and vocabulary for the understanding of the human body [88], while the second reflects how our life issues are transformed in biomedical ones with an emphasis on life sciences and technologies [89]. The increased knowledge on the epigenetic basis of disease and the possibility to revert aberrant epigenomes support the attention in internal determinants of the disease. In this context, it has to be underlined that the potential to revert the epigenome (e.g., by changing the lifestyle or by administrating a pill) could exacerbate the risk of “medicalization of life” instead of prevention.

### Genetic and epigenetic determinism in health

A third social trend that can also play a role in future health strategies is the widely accepted concept of genetic determinism. Genetic determinism derives from a misinterpretation of the influence of the genetic background on our phenotype and thus on our health or even on our behavior. Although it has been discredited and rebutted, the attractiveness of it postulates its apparent rationality which has been maintained in the collective imagination and has a great influence in the perception that society has of new health biotechnologies and their acceptance.

Moreover, genetic determinism can also appear in health when a non-Mendelian or multigenic genetic condition is diagnosed in an individual. In these cases, diagnosis is translated into a given probability of developing a disease and epigenetics may play a pivotal role in the final outcome and evolution of the disease. As we have already mentioned, epigenetics may be the only way to influence in this outcome. In spite of that, the perception of most people is driven by genetic determinism and they tend to believe in the inevitable that is written in genes.

Epigenetics has been proposed as the rationale to dodge genetic determinism in public opinion and daily life [90] but new strategies of modifying epigenetic profiles, either by changing lifestyles and environmental conditions or by developing epidrugs can add an overlay of epigenetic determinism on top of the already existing genetic determinism. To avoid this risk, communicative strategies that transmit the real potential of epigenetic interventions and their consequences should be developed.

## Conclusions

Current ELSI focused on epigenetic research has places upon the table new—or exacerbated old—concerns about the necessity of incorporating social concerns in basic and translational research. Reformulation of informed consents adapted to the complex epigenetic content, creating secure pathways to keep and share epigenetic data, or the equally access to health environments and heath care have been incorporated into the epigenetic language. Some of the ELSI mentioned before are similar to those associated with genetic research, but epigenetics adds a new dimension to be discussed in the ethics forum. How our social context (e.g., environmental exposition or lifestyle habits) could impact our health throughout epigenetic mechanism has markedly influenced the epigenetic discussion in ELSI and, especially, the potential risk of discrimination and stigmatization of vulnerable populations. In our opinion, caution should be exerted because robust and causal associations between those environmental factors and epigenetic changes are still pending. In contrast, it is clear that society should anticipate and develop an ethical reasoning from a multidisciplinary point of view. In addition, we strongly considered that a determinant concern in ELSI associated with epigenetics is communication. We strongly recommend the development of communication strategies to promote the individual ability to understand epigenetic relevant information and avoid the risks of prejudice and stigma as a tool for a better ethical assessment in epigenetic research. On the other hand, the education of ethical concerns within the scientific community needs to be promoted to guarantee an appropriate evaluation of the social consequences of epigenetic research. Finally, the engagement of the scientific community and the public policy makers will favor the development of decision models to finally implement epigenetic-based strategies in personalized medicine.

## Data Availability

Not applicable.

## Abbreviations

CpG: Cytosine-guanine dinucleotide
DNA: Deoxyribonucleic acid
ELSI: Ethical, legal and social issues
IHEC: International Human Epigenome Consortium
PAD: Patient decision aids
SNPs: Single-nucleotide polymorphisms
TCGA: The Cancer Genome Atlas
WGBS: Whole-genome bisulfite sequencing
